# Pig Movement and Antimicrobial Use Drive Transmission of Livestock-Associated Staphylococcus aureus CC398

**DOI:** 10.1128/mBio.02459-18

**Published:** 2018-12-11

**Authors:** Tara C. Smith, Meghan F. Davis, Christopher D. Heaney

**Affiliations:** a Kent State University College of Public Health, Kent, Ohio, USA; b Department of Environmental Health and Engineering, Johns Hopkins Bloomberg School of Public Health, Baltimore, Maryland, USA; c Department of Molecular and Comparative Pathobiology, Johns Hopkins School of Medicine, Baltimore, Maryland, USA; d Department of Epidemiology, Johns Hopkins Bloomberg School of Public Health, Baltimore, Maryland, USA; e Department of International Health, Johns Hopkins Bloomberg School of Public Health, Baltimore, Maryland, USA

**Keywords:** agriculture, antibiotics, *Staphylococcus aureus*, swine, antibiotic resistance

## Abstract

The epidemiology of methicillin-resistant Staphylococcus aureus has changed considerably over the last 3 decades, including the recognition of lineages associated with the community and with livestock exposure, in addition to nosocomial strains. A recent study by R. N. Sieber, R. L. Skov, J. Nielsen, J. Schulz, et al. (mBio 9:e02142-18, https://doi.org/10.1128/mBio.02142-18) demonstrates the importance of multisectoral cooperation at the intersection of occupational health, genomics, veterinary medicine practitioners, and farmers in order for us to better understand the epidemiology of antibiotic-resistant organisms.

## Abstract (precis)

The epidemiology of methicillin-resistant Staphylococcus aureus has changed considerably over the last 3 decades, including the recognition of lineages associated with the community and with livestock exposure, in addition to nosocomial strains. A recent study by R.

## COMMENTARY

The epidemiology of the opportunistic pathogen Staphylococcus aureus has changed substantially over the last 3 decades. S. aureus was originally considered chiefly a hospital-associated organism, but new classes of strains that emerged outside of the hospital setting were identified in the 1990s (community-associated methicillin-resistant S. aureus [CA-MRSA]) and in the 2000s (livestock-associated MRSA [LA-MRSA]). The latter was first associated with a single multilocus sequence type, ST398, and other closely related strains within clonal complex 398 (CC398). While CC398 strains were first identified in Europe (1, 2), they now are known to have a global distribution (3).

The application of whole-genome sequencing (WGS) has enabled better understanding of the potential origins of CC398 (4), and in the study discussed here, Sieber et al. (5) have applied this tool to better understand how animal movement within the Danish pig production system drives the spread of CC398. This work has important implications for control efforts to combat LA-MRSA within the Danish pig production system and may inform surveillance and intervention efforts globally.

Examining over 17,000 pig movements on 273 farms, Sieber and coauthors were able to determine that farms receiving animals from positive herds had a >4-fold-higher incidence rate than farms receiving animals from negative herds. They also noted that the dominant CC398 lineages (termed L1, L2, and L3 based on clustering within the phylogenetic tree) were those which contained multiple resistance genes, including those conferring resistance to aminoglycosides, lincosamides, and quinolones (antimicrobial drugs), as well as cadmium and zinc (metals). Finally, they demonstrated that the percentage of L1 to L3 cases in humans (skin and soft tissue infections [SSTI] and bloodstream infections [BSI]) also increased during the same temporal period, with L3 strains in particular demonstrating strong concordance with increases in pig prevalence and human SSTI (6).

This study’s integration of WGS and epidemiologic time-trend data from pigs and humans at a national level represents a significant knowledge advancement, beyond what has been achievable by typical studies in this field worldwide. Such studies typically employ less-granular molecular epidemiologic data (culture-based, PCR-based, or *spa* or multilocus sequence typing of S. aureus) collected from pigs or humans (not often from both), which can limit understanding of the relatedness and direction of transmission pathways. Although it is possible that the epidemic of LA-MRSA in humans in Denmark has been driven by factors besides pig movement and production activities, such as a common environmental reservoir for both people and animals or person-to-person transmission pathways, this study’s depth and breadth of epidemiologic and WGS data analysis from pig production networks and human carriage and infection isolates provide some of the strongest evidence to date of the relatedness of animal and human LA-MRSA carriage and infection isolates.

Of course, animal transportation has long been known to be a factor affecting the spread of disease, locally and internationally. The components of the 2009 H1N1 influenza virus appear to have been in place due to the international movement of swine (7). Early work suggested that importation of swine from Canada may have brought MRSA CC398 to the United States (8), though WGS methods were not employed to test this hypothesis. What is unique about this new work is the fine-grained detail provided about not only genomic sequences of bacteria isolated from the farms and the ability to track those from donor to recipient farms but also the possible ways that antimicrobial use on these farms may have influenced their survival and evolution. This includes uses of tetracycline and zinc supplementation of feed, which have previously been suggested to be drivers of MRSA evolution (9–11).

This work is one piece of a larger effort to identify how strains enter animal production (e.g., through pig breeding farms), disseminate through the production system, and ultimately expose humans through occupational or other animal contact, by residence in proximity to production facilities or fields impacted by animal manure, or via retail meat pathways. Sieber et al. comment that their WGS data suggest that early strains detected in the Danish production system were heterogeneous but that clonal expansion of a few strains led to the dominance of these lineages in the detected isolates in 2014 (5). Whether the early heterogeneity of strains reflects multiple introduction events onto breeding farms cannot be confirmed in the current work, but prior studies have suggested the potential for feed to play a role in the introduction of not just LA-MRSA strains (12) but also other animal pathogens (13). While the current work suggests that animal production systems—and animal movement within these systems—is a critical target for intervention, more work is needed to clarify the various upstream factors that likely contribute to introduction and reintroduction of LA-MRSA strains into the production chain.

Through identifying the importance of animal movement to the prevalence of LA-MRSA in the Danish pig production sector, this work highlights the critical importance of a One Health approach to understand and control anthropozoonotic pathogens. One Health approaches target not only human transmission pathways but also animal transmission networks, food production supply chains, and environmental reservoirs. Such efforts are inherently multidisciplinary and require careful harmonization of laboratory and data analysis methods (14). Given this, veterinary involvement and leadership will be needed in multiple sectors, from farm production through policy efforts, for intervention efforts to be successful and to prevent unintended consequences to animal health and well-being from implementation of these interventions. Equally, engagement of stakeholders in many related sectors, particularly those involving animal breeding and animal transportation and distribution, is essential to ensure that challenges that producers and distributors may face are addressed as part of any intervention effort.

As researchers endeavor to fill similar knowledge gaps in the United States, this study creates an exciting yet equally daunting prospect because it makes plain the resources and multisectoral cooperation that are needed to achieve knowledge advancements. What knowledge gains would be possible if similar or greater access and resources were available to answer these questions in pig and human populations in other leading global markets of pork production (e.g., North and South America and China)? This study serves as an excellent example of the important role of cross-sector cooperation whereby occupational health, public health, medical, veterinary, and agricultural researchers pursue studies with similar questions that are designed with an understanding of principles of molecular microbiology and epidemiology that must recognize how microbes know no borders, and that antimicrobial selective pressures and bacterial evolutionary patterns represent global public health challenges that will require our most advanced analytical and interdisciplinary cooperation to solve.
